# Seasonal variation in the incidence of primary intracerebral hemorrhage: a 16-year nationwide analysis

**DOI:** 10.3389/fneur.2023.1179317

**Published:** 2023-06-29

**Authors:** Eman Baig, Jonika Tannous, Thomas Potter, Alan Pan, Taya Prince, Gavin Britz, Farhaan S. Vahidy, Abdulaziz T. Bako

**Affiliations:** ^1^Department of Neurosurgery, Houston Methodist, Houston, TX, United States; ^2^Center for Health Data Science and Analytics, Houston Methodist, Houston, TX, United States; ^3^Department of Population Health Sciences, Weill Cornell Medical College, New York, NY, United States

**Keywords:** intracerebral hemorrhage, acute ischemic stroke, incidence rate, trends, seasonal variations

## Abstract

**Introduction:**

Data on nationwide trends and seasonal variations in the incidence of Intracerebral Hemorrhage (ICH) in the United States (US) are lacking.

**Methods:**

We used the Nationwide Inpatient Sample (2004–2019) and Census Bureau data to calculate the quarterly (Q1:January-March; Q2:April-June; Q3:July-September; Q4:October-December) incidence rates (IR) of adult (≥18 years) ICH hospitalizations, aggregated across Q1–Q4 and Q2–Q3. We report adjusted incidence rate ratios (aIRR) and 95% confidence intervals (CI) for differences in the quarterly incidence of ICH, as compared to acute ischemic stroke (AIS), between Q1Q4 and Q2Q3 using a multivariable Poisson regression model. We additionally performed stratified analyses across the four US regions.

**Results:**

Among 822,143 (49.0% female) ICH and 6,266,234 (51.9% female) AIS hospitalizations, the average quarterly crude IR of ICH was consistently higher in Q1Q4 compared to Q2Q3 (5.6 vs. 5.2 per 100,000) (aIRR, CI: 1.09, 1.08–1.11)—this pattern was similar across all four US regions. However, a similar variation pattern was not observed for AIS incidence. The incidence (aIRR, CI) of both ICH (1.01, 1.00–1.02) and AIS (1.03, 1.02–1.03) is rising.

**Conclusion:**

Unlike AIS, ICH incidence is consistently higher in colder quarters, underscoring the need for evaluation and prevention of factors driving seasonal variations in ICH incidence.

## Introduction

Primary Intracerebral Hemorrhage (ICH) is the second most common and most debilitating type of stroke accounting for 26% of all strokes globally (1) and 10% of all strokes in the United States (2). Seasonal variations in the incidence rate of ICH have been studied across the globe with largely inconsistent and conflicting findings (3–7). Specifically, some of these studies demonstrated the existence of significant seasonal variations in the incidence rate of ICH (3, 8, 9), with higher incidence rates reported in the colder months, and lower incidence rates reported in warmer months. In contrast, other studies indicated that seasonal variation does not exist for ICH (4, 6). Of note, nationwide data on seasonal fluctuations in the incidence of ICH across the United States have not been reported in the contemporary decade. Therefore, in this study, we evaluate trends and seasonal variations in ICH incidence and the association of age, sex, and race, with ICH incidence, across the United States between 2004 and 2019. We also utilized a comparison population of patients with acute ischemic stroke (AIS) and evaluated seasonal variations in AIS incidence. We hypothesized that ICH incidence will demonstrate significant seasonal variation across colder and warmer calendar quarters.

## Methods

### Ethics statement

The present study uses de-identified and publicly available data from the Healthcare Cost and Utilization Project (HCUP) (10) for analyses, and thereby meets the criteria for exempt research under our institution's policy. We followed the STrengthening the Reporting of OBservational studies in Epidemiology (STROBE) guidelines.

### Data availability

Qualified researchers can purchase the NIS data through the Health Care Utilization Project's central distributor (https://www.distributor.hcup-us.ahrq.gov/) upon completion of a data use agreement training.

### Data source, study design, and case identification

In a repeated cross-sectional design, adults (≥18 years) discharged with a principal diagnosis of ICH or AIS were identified from the Nationwide Inpatient Sample (NIS) between 2004 and 2019. NIS is the United State's largest all-payer in-hospital database representing over 90% of inpatient visits in the United States (11). We applied trend weights to pre-2012 NIS data to account for a sampling redesign that NIS underwent in 2012, making pre-2012 and post-2012 estimates comparable. Validated (12) International Classification of Disease (ICD), Ninth Revision and Tenth Revision codes were used to identify patients with ICH (ICD-9: 431; ICD-10: I61.0–I61.6 and I61.8–I61.9) and AIS (ICD-9: 433.x1, 434.x1, and 436; ICD-10: I63). Age was categorized as 18–44, 45–64, 65–74, and ≥75 years. Race/ethnicity was coded as non-Hispanic White, non-Hispanic Black, Asian American and Pacific Islanders, Hispanic, and Others (including Native Americans and Others). We excluded ICH patients with concurrent diagnoses of head trauma, arteriovenous malformations, intracranial aneurysms, and brain malignancy to focus our ICH sample on patients with primary non-traumatic ICH. Because the unit of observation in the NIS database is hospitalization, rather than individual patient, we excluded patients transferred to an acute care hospital to avoid double counting the same patient. We also excluded patients with missing age, sex, or race information. Similar to previous analyses (13), we obtained time-specific denominators for incidence rate calculation from the Census Bureau's public use microdata sample, based on the 1-year American community Survey (14).

### Statistical analysis

We aggregated ICH hospitalizations for each year into four discharge quarters: January–March (Q1); April–June (Q2); July–September (Q3) and October–December (Q4). Utilizing NIS discharge and trend weights, we calculated nationally representative estimates of the quarter-specific incidence of ICH and AIS hospitalizations across age, sex, and race/ethnicity sub-cohorts. Crude and adjusted per quarter incidence rates of ICH and AIS were calculated across colder (Q1Q4) and warmer quarters (Q2Q3). We used panel data Poisson regression model with random effects and robust standard errors to report the crude and adjusted incidence rate ratio (aIRR) and 95% confidence interval (CI) for the differences in the average quarterly incidence rate of ICH and AIS hospitalizations between Q1Q4 and Q2Q3. We additionally performed stratified analyses of seasonal differences in ICH incidence across the four US regions: Northeast, Midwest, South, and West. Also, to further assess the potential role of age in the observed seasonal variation in ICH incidence, we performed a stratified analysis among those aged <65 years and those aged ≥ 65 years. All analyses were conducted in STATA version 16. The alpha level of significance was set at 0.05.

### Sensitivity analyses

To evaluate the potential effect of the change in stroke incidence trend observed from 2007 to 2008, we performed sensitivity analyses, which excluded years prior to 2008.

## Results

**Figure 1:**
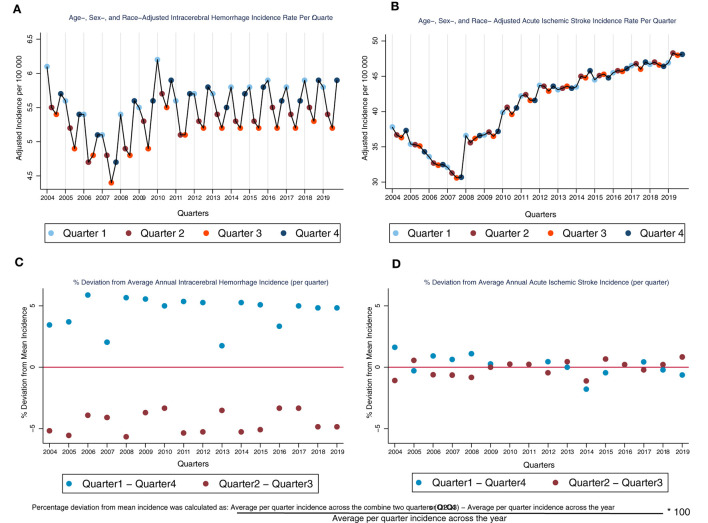
Age-, sex-, and race- adjusted incidence rate (per quarter) and percentage deviation from average annual incidence for ICH and AIS. Interpretative Key: Because the incidence rate of AIS is much higher than the rate for ICH, thereby making it difficult to visually compare of the graph for ICH and AIS **(A, B)**, we normalized the two graphs **(A, B)** by showing (in percentage points how the incidence rate of ICH deviates from the average quarterly incidence across the year **(C, D)**. In **(C)**, we see that the % deviation is positive for Q1Q4 and negative for Q2Q3 which indicates that the incidence rate is higher in Q1Q4 across all the years for ICH. However, if we compare this to the AIS graph **(D)**, we see that the % deviation is close to 0 for both Q1Q4 and Q2Q3, indicating that there is no or minimal seasonal fluctuation in AIS incidence through the years.

Across 16 years of NIS data, we identified 985,959 ICH and 7,067,178 AIS hospitalizations. Among these, 822,143 ICH and 6,266,234 AIS hospitalizations were included in the final analytic sample. A majority of the ICH patients were males (51%) and non-Hispanic White (65%), and approximately 64% of the ICH patients are aged ≥ 65 years (Table 1). In contrast, a majority of the AIS patients are females (52%). However, similar to the ICH population, most of the AIS patients are non-Hispanic White (70%) and aged ≥ 65 years (68%) (Table 1). Further details of the age, sex, and race distributions of AIS and ICH patients are provided in Table 1. The average quarterly crude incidence rate of ICH in Q1Q4 (5.6 per 100,000) was higher than the average rate of 5.2 per 100,000 observed in Q2Q3 (aIRR, CI: 1.09, 1.08–1.11), indicating a 9% higher ICH incidence in Q1Q4 compared to Q2Q3. The adjusted incidence rate of ICH was consistently higher in Q1Q4 (vs. Q2Q3) across all 16 years of data analyzed (Figure 1). Furthermore, we observed a similar pattern of higher ICH incidence in Q1Q4 (vs. Q2Q3) among all US regions [(Region: aIRR, CI) Northeast: 1.08, 1.06–1.10; Midwest: 1.09, 1.06–1.11; South: 1.10, 1.08–1.12; West: 1.09, 1.07–1.12] and among those aged <65 years and those aged ≥ 65 years [(Age-group: aIRR, CI) <65 years: 1.07, 1.06–1.09; ≥65 years: 1.11, 1.09–1.12]. For AIS, however, the average quarterly incidence in Q1Q4 (41.2 per 100,000) was similar to the incidence rate in Q2Q3 (41.2 per 100,000) (aIRR, CI: 1.00, 0.99–1.02). The incidence of both ICH (aIRR, CI: 1.01, 1.00–1.02) and AIS (aIRR, CI: 1.03, 1.02–1.03) significantly increased over time. Furthermore, the incidence rate of ICH was higher among non-Hispanic Blacks (aIRR, CI: 2.26, 1.90–2.68); Hispanic (aIRR, CI: 1.23, 1.10–1.38); Asian American and Pacific Islanders (1.42, 1.27–1.58) and the “Other” race/ethnicity category (aIRR, CI: 2.72, 2.39–3.10), compared to non-Hispanic Whites (Table 1). While non-Hispanic Blacks (aIRR, CI: 2.13, 1.82–2.48) and the “Other” race/ethnicity category (2.00, 1.80–2.22) had higher AIS incidence rate, compared to non-Hispanic Whites, the Asian American and Pacific Islanders had a lower AIS incidence rate than non-Hispanic Whites (aIRR, CI: 0.70, 0.64–0.77). We observed a progressive increase in the risk of both AIS and ICH with advancing age (Table 1).

**Table 1:** Factors associated with incidence rates of AIS and ICH hospitalizations.

	**ICH (*****n*** = **822,143)**		**AIS (*****n*** =**6,266,234)**	
**Variables**	***N* (%)**	**aIRR (95% CI)**	***N* (%)**	**aIRR (95% CI)**
Q1Q4 (vs. Q2Q3)	429,452 (52.24)	1.09 (1.08–1.11)^***^	3,135,267 (50.03)	1.00 (0.99–1.02)
Female	402,485 (48.96)	0.68 (0.65–0.71)^***^	3,253,451 (51.92)	0.79 (0.76–0.83)^***^
**Race**				
Non-Hispanic White	530,364 (64.51)	Reference	4,384,103 (69.96)	Reference
Non-Hispanic Black	142,274 (17.31)	2.30 (2.11–2.52)^***^	1,044,095 (16.66)	2.13 (1.97–2.30)^***^
Hispanic	78,330 (9.53)	1.22 (1.15–1.29)^***^	482,617 (7.70)	0.92 (0.88–0.96)^***^
Asian American and Pacific Islanders	39,648 (4.82)	1.38 (1.30–1.48)^***^	171,974 (2.74)	0.69 (0.65–0.73)^***^
Others	31,527 (3.83)	2.98 (2.77–3.21)^***^	183,445 (2.93)	2.08 (1.95–2.22)^***^
**Age**
18–44 years	50,836 (6.18)	Reference	260,833 (4.16)	Reference
45–64 years	249,015 (30.29)	8.09 (7.52–8.71)^***^	1,743,895 (27.83)	10.60 (10.01–11.23)^***^
65–74 years	172,379 (20.97)	18.56 (17.38–19.82)^***^	1,418,900 (22.64)	30.46 (28.86–32.15)^***^
≥75 years	349,913 (42.56)	45.70 (42.14–49.56)^***^	2,842,606 (45.36)	76.01 (70.93–81.47)^***^
Year^♣^	–	1.01 (1.00–1.01)^**^	–	1.03 (1.02–1.03)^***^

^**^Indicates *p*-value <0.01; ^***^indicates *p*-value <0.001.

^♣^The aIRR (95% confidence interval) for the year variable among ICH patients, rounded to three decimal figures is 1.005 (1.002–1.008).

All models were adjusted for age, sex, race/ethnicity and year of analysis.

### Sensitivity analysis

After restricting our years of analysis to 2008–2019, we found, similar to the main analyses, that ICH incidence is significantly higher in Q1Q4, compared to Q2Q3 (aIRR, CI: 1.09, 1.08–1.11), and AIS incidence in Q1Q4 was not significantly different from the incidence in Q2Q3 (aIRR, CI: 1.00, 0.98–1.02).

## Discussion

Across 16 years of nationwide data, our analyses demonstrate that the average per quarter incidence of ICH is consistently higher in the colder calendar months (Q1Q4), compared to the warmer months (Q2Q3). This difference was consistently significant across each of the 16 years analyzed and across all four United States census regions. However, we did not observe a similar pattern of seasonal variations in incidence among AIS patients.

To our knowledge, this is the first study to report nationwide seasonal variations in ICH incidence in the contemporary decade. The specific causes of seasonal fluctuations in ICH incidence are not fully understood; however, there are several potentially contributing mechanisms. For example, a recent meta-analysis demonstrated that blood pressure is higher during colder seasons of the year among both normotensive and hypertensive patients (15). Exposure to colder weather may therefore contribute to variation in ICH incidence via such hemodynamic changes (16). Other potential explanations for the observed variations in ICH incidence may include holiday stress—which may contribute to changes in health behaviors, such as diet, physical activity, and medication adherence—as well as seasonal changes in biochemical markers, including blood viscosity, serum fibrinogen, and cholesterol levels (17, 18). Furthermore, although AIS and ICH largely share common predisposing factors, ICH, compared to AIS, is more sensitive to acute changes in blood pressure, which is widely believed to be the most important risk factor for ICH (19–21). On the other hand, the risk of AIS has been demonstrated to be largely driven by atherosclerotic processes (21–23). It is likely that acute changes in health behavior influence vascular dynamics altering blood pressure. In contrast, the effect of health behaviors on the advancement of atherosclerosis to critical levels may be more insidious. Therefore, the fact that blood pressure significantly increases during colder seasons of the year may explain why significant seasonal variations were only observed for ICH incidence, and not for AIS incidence (15, 24–27). Overall, the mechanistic basis of higher ICH incidence across particular seasons needs to be further investigated and will directly contribute to developing risk-specific preventive strategies.

Furthermore, we found a similar pattern of seasonal variation in ICH incidence across all four regions of the United States, further corroborating the findings of a recent meta-analysis, which showed that regardless of a region's climate characteristics, ICH incidence is higher in colder seasons, compared to warmer seasons, of the year (3). Moreover, the existence of seasonal variation in ICH incidence across all four regions of the United States potentially reflects the consequence of the globally reported rise in blood pressure during colder seasons (15, 24–27). We would also like to state that a more granular categorization of geographic regions based on climate characteristics in lieu of the census regions provided by NIS may be necessary to study the effect of seasonal temperature variations on ICH incidence.

Our data also indicates that the incidence of both ICH and AIS are rising over time, reinforcing the need to further strengthen stroke prevention programs in the US. Also, similar to previous reports (2, 28), we found that the incidence rates of both ICH and AIS are higher among males and non-Hispanic Blacks (vs. non-Hispanic Whites). While a higher prevalence of poorly controlled stroke risk factors among these sociodemographic subpopulations may explain the observed differences in incidence rate (12), further evaluation of the epidemiological underpinnings of these differences is warranted.

Potential study limitations include the utilization of ICD codes for ICH and AIS identification rather than neuroimaging, which may lead to misclassification bias. However, ICD codes are highly accurate in identifying acute stroke cases (27). Our analysis may have missed non-hospitalized stroke cases, but given that 95% of acute stroke cases are followed by immediate hospitalization (29), our methodology complies with the Health Care Utilization Project's recommendation for IR estimation using NIS (28, 29). Finally, the potential inclusion of recurrent AIS and ICH hospitalizations may lead to overestimation of AIS and ICH incidence rates. However, the 1-year risk of AIS and ICH have been reported to be <10 and 5%, respectively (30, 31).

## Conclusions

Unlike AIS, ICH hospitalizations consistently demonstrate significant seasonal variations, with higher rates observed in colder quarters, compared to warmer quarters. Our findings call for an evaluation of the factors contributing to seasonal variations in ICH incidence and underscore the need for season-specific preventive measures to reduce the incidence of intracerebral hemorrhage.

## Data availability statement

The data analyzed in this study was obtained from the Agency for Healthcare Research and Quality (AHRQ) Health Care Utilization Project (HCUP) Central Distributor (https://www.distributor.hcupus.ahrq.gov/), the following licenses/restrictions apply: Qualified researchers can purchase the Nationwide Inpatient Sample (NIS) data upon completion of a Data Use Agreement (DUA) training. Requests to access these datasets should be directed to AHRQ HCUP Central Distributor, hcup@ahrq.gov.

## Ethics statement

Ethical review and approval was not required for the study on human participants in accordance with the local legislation and institutional requirements. Written informed consent for participation was not required for this study in accordance with the national legislation and the institutional requirements.

## Author contributions

EB and AB: drafting/revision of the manuscript, study concept and design, and analysis and interpretation of data. JT, TPo, AP, and TPr: critical revision of the manuscript, study concept and design, and interpretation of data. GB: critical revision of the manuscript for content and major role in the acquisition of data. FV: drafting/revision of the manuscript for content, study concept and design, and analysis and interpretation of data. All authors contributed to the article and approved the submitted version.

## References

[1] Krishnamurthi RV Ikeda T Feigin VL. Global, regional and country-specific burden of ischaemic stroke, intracerebral haemorrhage and subarachnoid haemorrhage: a systematic analysis of the Global Burden of disease study 2017. Neuroepidemiology. (2020) 54:171–9. 10.1159/000506396 32079017

[2] Tsao CW Aday AW Almarzooq ZI Alonso A Beaton AZ Bittencourt MS. Heart disease and stroke statistics-2022 update: a report from the American Heart Association. Circulation. (2022) 145:e153–639. 10.1161/CIR.0000000000001052 35078371

[3] Kuzmenko NV Galagudza MM. Dependence of seasonal dynamics of hemorrhagic and ischemic strokes on the climate of a region: a meta-analysis. Int J Stroke. (2022) 17:226–35. 10.1177/17474930211006296 33724111

[4] Oberg AL Ferguson JA McIntyre LM Horner RD. Incidence of stroke and season of the year: evidence of an association. Am J Epidemiol. (2000) 152:558–64. 10.1093/aje/152.6.558 10997546

[5] Lanska DJ Hoffmann RG. Seasonal variation in stroke mortality rates. Neurology. (1999) 52:984–90. 10.1212/WNL.52.5.984 10102417

[6] Jin H Xu Z Li Y Xu J Shan H Feng X. Seasonal variation of stroke incidence in Wujin, a city in southeast China. Health Sci Rep. (2018) 1:e29. 10.1002/hsr2.29 30623065 PMC6266434

[7] Raj K Bhatia R Prasad K Srivastava MVP Vishnubhatla S Singh MB. Seasonal differences and circadian variation in stroke occurrence and stroke subtypes. J Stroke Cerebrovasc Dis. (2015) 24:10–6. 10.1016/j.jstrokecerebrovasdis.2014.07.051 25284717

[8] Inagawa T. Diurnal and seasonal variations in the onset of primary intracerebral hemorrhage in individuals living in Izumo City, Japan. J Neurosurg. (2003) 98:326–36. 10.3171/jns.2003.98.2.0326 12593619

[9] Capon A Demeurisse G Zheng L. Seasonal variation of cerebral hemorrhage in 236 consecutive cases in Brussels. Stroke. (1992) 23:24–7. 10.1161/01.STR.23.1.24 1731416

[10] NIS, Database Documentation. The Healthcare Cost and Utilization Project. Available online at: https://www.hcup-us.ahrq.gov/db/nation/nis/nisdbdocumentation.jsp (accessed February 2, 2023).

[11] Fingar KR, Owens, PL, Barrett, ML, Steiner, CA,. Using the HCUP Databases to Study Incidence Prevalence. HCUP Methods Series Report # 2016-06 ONLINE US Agency for Healthcare Research Quality (2016). Available online at: https://www.hcup-us.ahrq.gov/reports/methods/2016-06.pdf (accessed August 7, 2021).

[12] McCormick N Bhole V Lacaille D Avina-Zubieta JA. Validity of diagnostic codes for acute stroke in administrative databases: a systematic review. PLoS ONE. (2015) 10:e0135834. 10.1371/journal.pone.0135834 26292280 PMC4546158

[13] Bako AT Pan A Potter T Tannous J Johnson C Baig E. Contemporary trends in the nationwide incidence of primary intracerebral hemorrhage. Stroke. (2022) 53:e70–4. 10.1161/STROKEAHA.121.037332 35109682

[14] American Community Survey Public Use Microdata Sample. Census Bureau. Available online at: https://data.census.gov/mdat/#/ (accessed July 1, 2021).

[15] Kollias A Kyriakoulis KG Stambolliu E Ntineri A Anagnostopoulos I Stergiou GS. Seasonal blood pressure variation assessed by different measurement methods: systematic review and meta-analysis. J Hypertens. (2020) 38:791–8. 10.1097/HJH.0000000000002355 32102047

[16] Thrift AG McNeil JJ Forbes A Donnan GA. Risk factors for cerebral hemorrhage in the era of well-controlled hypertension. Stroke. (1996) 27:2020–5. 10.1161/01.STR.27.11.2020 8898809

[17] Turin TC Kita Y Murakami Y Rumana N Sugihara H Morita Y. Higher stroke incidence in the spring season regardless of conventional risk factors. Stroke. (2008) 39:745–52. 10.1161/STROKEAHA.107.495929 18258821

[18] Tofler GH Muller JE. Triggering of acute cardiovascular disease and potential preventive strategies. Circulation. (2006) 114:1863–72. 10.1161/CIRCULATIONAHA.105.596189 17060396

[19] Fischer U Cooney MT Bull LM Silver LE Chalmers J Anderson CS. Acute post-stroke blood pressure relative to premorbid levels in intracerebral haemorrhage versus major ischaemic stroke: a population-based study. Lancet Neurol. (2014) 13:374–84. 10.1016/S1474-4422(14)70031-6 24582530 PMC4238109

[20] Wajngarten M Silva GS. Hypertension and stroke: update on treatment. Eur Cardiol. (2019) 14:111–5. 10.15420/ecr.2019.11.1 31360232 PMC6659031

[21] Tsao CW Aday AW Almarzooq ZI Anderson CAM Arora P Avery CL. Heart disease and stroke statistics-2023 update: a report from the American Heart Association. Circulation. (2023) 147:e93–e621. 10.1161/CIR.0000000000001123 36695182 PMC12135016

[22] Ekker MS Verhoeven JI Schellekens MMI Boot EM van Alebeek ME Brouwers PJAM. Risk factors and causes of ischemic stroke in 1322 young adults. Stroke. (2023) 54:439–47. 10.1161/STROKEAHA.122.040524 36511150 PMC9855752

[23] Silva GS Koroshetz WJ González RG Schwamm LH. Causes of ischemic stroke. In: Acute Ischemic Stroke. Berlin: Springer Berlin Heidelberg (2011). p. 25–42.

[24] Stergiou G. S Palatini P Modesti P. A. Asayama K. Asmar R Bilo G. Seasonal variation in blood pressure: evidence, consensus and recommendations for clinical practice Consensus statement by the European Society of Hypertension Working Group on Blood Pressure Monitoring and Cardiovascular Variability. J Hypertens. (2020) 38:1235–43. 10.1097/HJH.0000000000002341 31990898

[25] Narita K Hoshide S Kario K. Seasonal variation in blood pressure: current evidence and recommendations for hypertension management. Hypertens Res. (2021) 44:1363–72. 10.1038/s41440-021-00732-z 34489592

[26] Rosenthal T. Seasonal variations in blood pressure. Am J Geriatr Cardiol. (2004) 13:267–72. 10.1111/j.1076-7460.2004.00060.x 15365290

[27] Wang Q Li C Guo Y Barnett AG Tong S Phung D. Environmental ambient temperature and blood pressure in adults: a systematic review and meta-analysis. Sci Total Environ. (2017) 575:276–86. 10.1016/j.scitotenv.2016.10.019 27750133

[28] Gardener H Sacco RL Rundek T Battistella V Cheung YK Elkind MSV. Race and ethnic disparities in stroke incidence in the Northern Manhattan Study. Stroke. (2020) 51:1064–9. 10.1161/STROKEAHA.119.028806 32078475 PMC7093213

[29] Sacco RL Boden-Albala B Gan R Chen X Kargman DE Shea S. Stroke incidence among white, black, and Hispanic residents of an urban community: the Northern Manhattan Stroke Study. Am J Epidemiol. (1998) 147:259–68. 10.1093/oxfordjournals.aje.a009445 9482500

[30] Dhamoon MS Sciacca RR Rundek T Sacco RL Elkind MSV. Recurrent stroke and cardiac risks after first ischemic stroke: the Northern Manhattan Study. Neurology. (2006) 66:641–6. 10.1212/01.wnl.0000201253.93811.f6 16534100

[31] Leasure AC King ZA Torres-Lopez V Murthy SB Kamel H Shoamanesh A. Racial/ethnic disparities in the risk of intracerebral hemorrhage recurrence. Neurology. (2020) 94:e314–22. 10.1212/WNL.0000000000008737 31831597 PMC7108806

